# How Do You Fill Pulp Canals?

**Published:** 1905-04-15

**Authors:** A. W. Harlan

**Affiliations:** New York


### HOW DO YOU FILL PULP CANALS?

BY A. W. HARLAN, M.D., D.D.S., NEW YORK.

Editor Dental Register—Dear Sir: Since my removal to New York city, and engaging the practice of treating pyorrhœa, I have so many times met cases which would not respond to any treatment, that I have come to suspect every tooth having a root-canal filling. After removing the deposits on the roots and cutting away the necrosed bone and disinfecting in the usual manner I have in a number of cases been confronted with a persistent discharge of pus along the sides of the roots. I have opened into enough such canals to convince me that in all such cases we can almost certainly conclude that we have a faulty root-canal filling and an apical abscess to contend with. Examination of these cases with a powerful electric light, containing the actinic ray, will generally prove the correctness of such a diagnosis. I have opened into the canals of the single as well of the multiple-rooted teeth and find not much difference as to the thoroughness with which they are filled. I have found a great variety of filling materials in these roots, such as cottonwood, asbestos, copper wire, whalebone, etc., and all within a short period of one year. I have been compelled to disinfect, treat and refill these roots canals before I could complete the treatment of the gums and peridental structures. Now what I want to know is why there is so much uncertainty in successful treatment of pulpless teeth? Why so great variety in materials, lack of thoroughness in manipulation as to lead to such disastrous results? If the pulp is thoroughly removed and the canal is made surgically clean, why can we not fill the canal with some material that will certainly and definitely close the apical opening, and thus prevent recurrent injection, which is so likely to occur from the use of such filling, or stuffing materials as are noted above? It has been said that gutta-percha root filling can not be depended upon to close the apical opening and that when removed from the canals after several months they always indicate by the odor brought with them that decomposition has taken place in the canal. I suspect in all such cases that the fault is not so much with the material as with the person using it. In my experience I have found that every trace of organic material must be removed from the canal before it is closed with the filling, or there is bound to be infection or recurrent disease. The pulp tissue must be destroyed chemically and washed out to the apex of the root, and the canal thoroughly sterilized or no material will seal it hermetically and prevent incipient or recurrent disease. In my estimate of this question I am led to believe that too little time and well-directed—and often laborious—effort has been expended upon this work, especially in respect to the thorough removal of the pulp remnants, surgically and chemically, and to the preliminary disinfection before the root-filling is introduced. The preparation of the canal for the material used is not thoroughly made and the manipulation itself is faulty. To-day I opened two canals that had been well prepared, but were filled not over two-thirds of their lengths. I do not know how a root-canal that is at all constricted or tortuous can be filled to its apex with wire, or cotton, or whalebone, or any such substance. In such roots the material must be of such a character that it will flow under proper pressure to the end of the roots through any conceivable canal, however tortuous. Can you to your readers enlighten me on this subject?

———

The communication of Dr. Harlan opens up a question that has been discussed so much that it seems thread-bare. Yet the application to the special work which he is doing makes it one of great interest to him at least, and calls attention again to the fact that the practice of root-filling is not as perfectly understood or executed as it can and should be. We have been looking for this subject to come to the front, but from a different source or cause. The use, or rather abuse, of the much extolled abscess cures and pulp mummifiers is bound to bring on sooner or later an enormous crop of "gum boils." We have been so long reminded of the many substances that can be used for stopping root-canals, that we are not surprised to learn that Dr. Harlan has found so great a variety in his field of practice. Being located in a great city and seeing patients from not only his city, but from all the country, he would be more likely to encounter a greater variety of methods of practice than the average general practitioner. We are a little surprised that he should encounter the cotton root-filling, as we supposed no one, in this enlightened age, would risk the welfare of his patients with a material so universally condemned. Since the advent of the easy and painless methods of pulp extirpation have been so universally practiced, we imagine that the preservation, or attempted preservation, of the tooth pulp when uncovered by disease, has become practically a lost art. At least it is not artistically practiced to any considerable extent. The propagation of the doctrine that the tooth pulp is a useless organ after the tooth is fully formed, and that its vitality is a matter of no consequence so far as the future value or function of the tooth is concerned, has also had much to do with the reckless destruction of this very valuable organ, added to this, the other proposition that the most careful removal of all possible organic tissue from the canal is not important or essential so long as a comparatively-indestructible antiseptic root-filling or mummifier is sealed into the canal. This latter factor has had much to do with the promoting of "sure cure" and "never fail" secret nostrums by the supply houses and their adoption by ignorant and lazy practitioners, with consequent result that there is probably more careless work done in pulp canals than in any other branch of dental practice. There must come a reaction, and the sooner it comes the better it will be for us. It seems to us that the question of selecting a suitable root-canal filling material is not a practicable impossibility. The researches of Dr. Webster, as recorded in the 1903 *Dental Review*, pages 275 and 342, would indicate that the materials most generally relied upon for root fillings, cement and gutta-perchas, as well as many others are not capable of so sealing a root-canal as to prevent the ingress of bacteria. We have no time or place here to record his research even in an epitomized manner, but can only say that a long history of using these materials by careful clinicians has resulted in their approval as the most useful materials known at this time for the conservative treatment of pulpless teeth, and this history will controvert the findings of Dr. Webster's research until such time as he can devise a method of conducting his research along lines more analagous to the conditions which prevail in the teeth. We think Dr. Harlan intimates, if he does not actually state, the cause for failure of root-canal fillings to prevent apical abscess. It is more often the lack of skill in the operator than fault with the materials used. In our experience we have found that gutta-percha can be more exactly manipulated in the majority of cases than any other material. It is almost entirely indestructible and can be made sufficiently flexible to permit its introduction into small and even tortuous canals. We know several conscientious operators who are skillful enough to manipulate the oxy-chloride of zinc so well that they have practically and universally successful results from its use as a root-filling. If roots are properly prepared by surgical manipulation and as conscientiously sterilized and filled with either gutta-percha or oxy-chlorid of zinc to the apical foramen the chances of alveolar abscess will be so small as to make the method and materials all that can be desired.

Dr. Otto Ingliss, in his work on Dental Pathology and Therapeutics, gives the following qualities which a root-canal filling material should possess. It should be non-irritant and hermetically seal the canal, and should be unalterable in the conditions which surround it. It should have a continuous antiseptic reaction and be easily removed in case of necessity. These qualities are found in gutta-percha into which some durable antiseptic has been incorporated. This material can be adapted to a canal which has been reamed and enlarged so as to permit of easy introduction of the gutta-percha which may be manipulated by softening it with heat or liquifying it with chloroform to such a consistence that it can be forced into a tortuous canal and made to permeate every part of any irregular surfaces. When liquified it is best manipulated in connection with a hard gutta-percha cone which reduces to a minimum the shrinkage from the solvent being absorbed or evaporated. To secure the best results the canal should be thoroughly prepared surgically and disinfected. Before introducing the filling the canal should be washed out with alcohol and dehydrated with the hot-air syringe or root-dryer, and then saturated with eucalyptus oil. The chlora-percha is then introduced by means of a suitable probe on which a few shreds of cotton are wound to serve as a carrier and the chloro-percha can then be carried to the end of the root and the walls of the canal coated so that the gutta-percha cone will glide readily to its position. A hard gutta-percha cone is attached to a root-canal plugger which has been slightly warmed so that it will adhere to the cone, the heat softens the cone enough to allow it to bend and follow the tortuous canal. The gutta-percha cone should not fill more than one-third of the apical end of the root, leaving the balance to be filled with oxy-chloride of zinc cement, and the crown cavity with such more durable filling as may be desired. This method securely stops the apical foramen and the zinc cement acts as an antiseptic filling, especially when some strong or durable antiseptic has been incorporated with the cement when mixed.

Dr. Elgin MaWhinney, in the newest work on oral pathology and therapeutics, just off the press, treats this subject in much the same manner and we give here a brief synopsis of his chapter on the preparation and filling of root-canals. After describing the anatomy of the different canals and the methods of removing the pulps, he gives particular instruction as to methods of performing the surgical part of the process under strictly-aseptic conditions, and points out the great importance of exceeding care that this shall be absolutely accomplished. He advocates the use of sulphuric acid to cleanse and enlarge the canal and loosen the nodular deposits, also the pulp digestors, such as carica papaya, dissolved in slightly-acidulated water, which he would seal in the canal for from ten to twenty days. For the pulp mummifiers he says he "*has no respect.*" He says of "immediate root-filling," if the work of removing the vital pulp has been thoroughly done, with all cautions against infection, it would seem advisable, after bathing the canal with some mild antiseptic, such as a mixture of eucalyptus, cloves and trikresol, and drying it out with alcohol. It is, however, a more definite practice to allow the antiseptic solution to remain in the canal for a few days before filling. No subject has received more attention during the last fifteen years than the process of filling root-canals, and no ideal filling has yet been found. The ideal root-canal filling should not be porous, irritating or shrink after it is placed, and should be readily manipulated and capable of being placed accurately in the most constricted or tortuous canals. We have no such material at the present time. The one which most nearly qualifies at the present time is gutta-percha, and this is probably more used than any other material. To use gutta-percha for large canals, select first a small cone that will fit and fill the apical foramen as nearly as this can be determined by testing with the probe. Moisten the canal with eucalyptus after it has been sterilized and made dry, then place the sterilized gutta-percha cone on the end of a warm canal plugger and force this piece to place so that it fills completely the apical foramina. Then fill the rest of the canal with suitable pieces of gutta-percha using the hot-air syringe to soften the gutta-percha in the canal so that it may be the more solidly packed. In small canals select a small cone that is stiff enough to be forced to the end of the root. The canal is moistened as before with eucalyptus and liquified gutta-percha is then pumped into the canal with a suitable probe or brooch wound with a shred of cotton. Care should be taken to insure the forcing of the chloro-percha to the end of the root in these canals by continued pumping. Then insert the small stiff gutta-percha cone and gently but firmly force it to the apical foramina and leave it there as a seal. The excess of chloro-percha should be allowed to flow back in the canal rather than be forced through the foramina. When the canals are very small, finely-pointed gold or copper cones can be used in place of the gutta-percha, as they will carry to the end of the canal more definitely. In the larger canals, where gutta-percha cones are used, they should be placed as nearly as practicable and then the chloro-percha evaporated until all shrinkage has taken place, when the gutta-percha can be warmed and repacked. The object to be attained in all cases is to pack the canal to the end with the gutta-percha and any method that individual skill may devise for accomplishing this end certainly should be used. There will always be a variation in exact manipulation corresponding with the variety in one's habit of using instruments. There may also be some variation in materials to suit peculiar idiosyncracies. A delicate tactile sense, natural or acquired, will be a large factor in the success of this operation from the manipulative aspect. The materials and method described will serve to illustrate the general practice of the more conservative operators, and, when carefully made, will produce satisfactory results.

